# A Functional Screen Provides Evidence for a Conserved, Regulatory, Juxtamembrane Phosphorylation Site in Guanylyl Cyclase A and B

**DOI:** 10.1371/journal.pone.0036747

**Published:** 2012-05-09

**Authors:** Andrea R. Yoder, Jerid W. Robinson, Deborah M. Dickey, Joshua Andersland, Beth A. Rose, Matthew D. Stone, Timothy J. Griffin, Lincoln R. Potter

**Affiliations:** 1 Department of Pharmacology, University of Minnesota Medical School, Minneapolis, Minnesota, United States of America; 2 Department of Biochemistry, Molecular Biology and Biophysics, University of Minnesota, Minneapolis, Minnesota, United States of America; Indiana University School of Medicine, United States of America

## Abstract

Kinase homology domain (KHD) phosphorylation is required for activation of guanylyl cyclase (GC)-A and -B. Phosphopeptide mapping identified multiple phosphorylation sites in GC-A and GC-B, but these approaches have difficulty identifying sites in poorly detected peptides. Here, a functional screen was conducted to identify novel sites. Conserved serines or threonines in the KHDs of phosphorylated receptor GCs were mutated to alanine and tested for reduced hormone to detergent activity ratios. Mutation of Ser-489 in GC-B to alanine but not glutamate reduced the activity ratio to 60% of wild type (WT) levels. Similar results were observed with Ser-473, the homologous site in GC-A. Receptors containing glutamates for previously identified phosphorylation sites (GC-A-6E and GC-B-6E) were activated to ∼20% of WT levels but the additional glutamate substitution for S473 or S489 increased activity to near WT levels. Substrate-velocity assays indicated that GC-B-WT-S489E and GC-B-6E-S489E had lower Km values and that WT-GC-B-S489A, GC-B-6E and GC-B-6E-S489A had higher Km values than WT-GC-B. Homologous desensitization was enhanced when GC-A contained the S473E substitution, and GC-B-6E-S489E was resistant to inhibition by a calcium elevating treatment or protein kinase C activation – processes that dephosphorylate GC-B. Mass spectrometric detection of a synthetic phospho-Ser-473 containing peptide was 200–1300-fold less sensitive than other phosphorylated peptides and neither mass spectrometric nor ^32^PO_4_ co-migration studies detected phospho-Ser-473 or phospho-Ser-489 in cells. We conclude that Ser-473 and Ser-489 are Km-regulating phosphorylation sites that are difficult to detect using current methods.

## Introduction

Natriuretic peptides are endogenous disulfide-linked endocrine and paracrine molecules, and recombinant forms of these peptides are drugs approved for the treatment of congestive heart failure [1], [2]. Atrial natriuretic peptide (ANP) and B-type natriuretic peptide regulate the cardiovascular system by activating guanylyl cyclase (GC)-A, whereas C-type natriuretic peptide (CNP) regulates long bone growth and meiotic resumption in oocytes by activating GC-B [3]. Both GC-A and GC-B are membrane GCs that contain a large extracellular ligand-binding domain, a single membrane span and intracellular kinase homology domain (KHD), dimerization domain, and C-terminal GC domain [4], [5]. Both GC-A and GC-B signal by catalyzing the synthesis of cGMP in response to natriuretic peptide binding.

**Figure 1:**
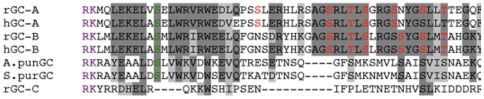
Comparison of the N-terminal region of the kinase homology domains of transmembrane guanylyl cyclase receptors. Purple RK indicates the beginning of intracellular domains. Red residues are confirmed phosphorylation sites. The green residue is a putative conserved phosphorylation site, which is Ser-489 in GC-B and Ser-473 in GC-A. Sequences were aligned with CLUSTAL version 2.0.1. Abbreviations are: rGC-A, rat GC-A; hGC-A, human GC-A; rGC-B, rat GC-B; hGC-B, human GC-B; hGC-C, human GC-C; A. punGC, GC from sea urchin species A. punctalata; S. purGC, GC from sea urchin species S. purpuratus.

Phosphorylation of the KHD is required for the activation of GC-A and GC-B and dephosphorylation reduces the GC activity of these receptors [6], [7], [8], [9], [10]. Seven phosphorylation sites (Ser-487, Ser-497, Thr-500, Ser-502, Ser-506, Ser-510 and Thr-513) were identified in GC-A and six phosphorylation sites (Ser-513, Thr-516, Ser-518, Ser-523, Ser-526 and Thr-529) were identified in GC-B (Figure 1) [11], [12], [13], [14]. Single serine or threonine to alanine substitutions for all sites with the exception of Ser-487 in GC-A reduced receptor phosphate content and natriuretic peptide-dependent GC activity without affecting protein levels or detergent-dependent GC activity [12]. Mutation of Ser-497, Thr-500, Ser-502, Ser-506 and Ser-510 in GC-A or all six sites in GC-B to alanine resulted in receptors that could not be activated by natriuretic peptides, whereas mutation of the first six identified sites in either receptor to glutamate produced receptors that were activated by natriuretic peptides but to a lessor extent than the WT receptors [11], [12], [14], [15].

The role of phosphorylation of Ser-487 in regulation of GC-A is controversial. Mutation of this site to glutamate decreased activity but mutation to alanine had no effect. Schroter et al. found that a Ser-487-Glu mutant failed to desensitize, whereas our group found that receptors containing alanine or glutamate substitutions for Ser-487 desensitized similarly to WT-GC-A [13], [14].

Although many phosphorylation sites have been found in GC-A and GC-B, several observations support the notion that undiscovered phosphorylation sites exist in these receptors. First, highly homologous, peptide-activated sea urchin GCs, which are also desensitized by dephosphorylation, contain 15 to 17 moles of phosphate/receptor [16], [17]. Secondly, only about 10% of the GC-A or GC-B tryptic phosphopeptides were recovered after purification [18]. Thirdly, conversion of all known phosphorylation sites to glutamates yielded enzymes that are only about 20% as active as the WT phosphorylated receptors [14], [15]. Fourth, tryptic phosphopeptide maps of GC-A and GC-B isolated from cells treated in the presence or absence of natriuretic peptide are identical even though this treatment causes a 50% reduction in total receptor phosphate content [8], [19]. Several attempts were made to identify additional sites by classic ^32^PO_4_ phosphopeptide mapping techniques or by mass spectrometry [11], [12], [13], [14], but these efforts were limited by the ability to purify or detect proteolytic peptides containing the novel site or sites.

The goal of this study was to identify undiscovered natriuretic peptide receptor phosphorylation sites using a functional screen that was independent of phosphopeptide purification or detection. A highly conserved juxtamembrane serine was identified in membrane GCs that are regulated by phosphorylation. Alanine but not glutamate substitutions for this residue in GC-A and GC-B reduced natriuretic peptide-dependent, but not detergent dependent, GC activity. Substrate-velocity assays indicated that GC-B activity losses associated with the alanine substitutions were due to large increases in the Michaelis constant and smaller decreases in the maximal velocity. Inhibition studies revealed that GC-A receptors containing a glutamate for Ser-473 exhibited enhanced homologous desensitization and that the dephosphorylation resistant receptor, GC-B-6E-S489E, was not inhibited by a calcium elevating treatment or protein kinase C activation.

**Table 1:** The effect of alanine substitutions for known or potential GC-B phosphorylation sites on guanylyl cyclase activity and indication of conservation of sites in GC-A and sea urchin guanylyl cyclases is shown.

*Residue*	*Cyclase reduction*	*Conserved in GC-A*	*Conserved in sea urchin*
*Known sites*			
S513	yes	yes	no
T516	yes	yes	yes
S518	yes	yes	no
S523	yes	no	yes
S526	yes	yes	yes
T529	yes	yes	yes
*Potential sites*			
S489	yes	yes	yes
S504	no	no	yes
S647*	yes *	yes	yes
S654	no	no	no
T661	no	yes	yes
S667	no	yes	no
S677	no	no	no
T687	no	yes	yes
S721	no	yes	no

* Indicates alanine substitution reduced guanylyl cyclase activity but receptor was determined to be incompletely glycosylated and phosphorylated.

## Results

### Initial screen of potential intracellular phosphorylation sites

To identify conserved serines or threonines, a multiple sequence alignment of the intracellular region of membrane GCs across several species was performed (Figure 1). The GCs compared were rat GC-A, human GC-A, rat GC-B, human GC-B, and the sea urchins species *A. punctulata* and *S. purpuratus*, as well as rat GC-C. The first six GCs are regulated by phosphorylation whereas GC-C lacks the phosphorylation sites identified in GC-A and GC-B [11], [12], [16], [20]. Therefore, we expected that serines and threonines conserved in the top six GCs but not in GC-C would be potential phosphorylation sites in GC-B.

Targeted residues were initially mutated to alanine in the rat GC-B cDNA and expression, glycosylation and phosphorylation of the receptors were verified by Western blotting (Figure S1). Three distinct species of the WT receptor were observed by Western blot: a thin, fastest migrating (lowest molecular weight) unglycosylated species, an intermediately migrating glycosylated species and a slowest migrating (highest molecular weight) fully-glycosylated species (arrow) that is maximally phosphorylated and hormonally responsive [7], [11]. Alanine substitution for Ser-647 alone or in combination with 648 resulted in the loss of the fully processed receptor, whereas none of the other mutations affected GC-B processing. Similar improper receptor expression and processing likely explains why alanine substitutions for the homologous two serines in GC-A resulted in a receptor that was not activated by ANP [21].

GC activity was measured in membranes from 293 cells transfected with WT or mutant receptors in the presence of natriuretic peptide, ATP and magnesium-GTP or in the presence of 1% Triton X-100 and manganese-GTP. Analysis of previously identified phosphorylation sites in GC-A and GC-B under these conditions found that the alanine substitutions reduced natriuretic peptide-dependent activity but not detergent-dependent activity [11], [12]. Therefore, activities were expressed as a ratio of natriuretic peptide-dependent activity to detergent-dependent activity to control for differing transfection efficiencies and expression levels [15]. The ratio from each mutant was then compared to the ratio of the WT receptor (Table 1). If the mutant activity ratio was reduced, then that site was considered a candidate for further evaluation. Besides the Ser-647 substitution that blocked receptor processing, only one mutation, Ser-489, reduced the GC activity ratio. Alanine substitutions at Ser-504, Ser-654, Thr-661, Ser-667, Ser-677, Thr-687 and Ser-721 failed to reduce the activity ratio of GC-B and were not further evaluated.

**Figure 2:**
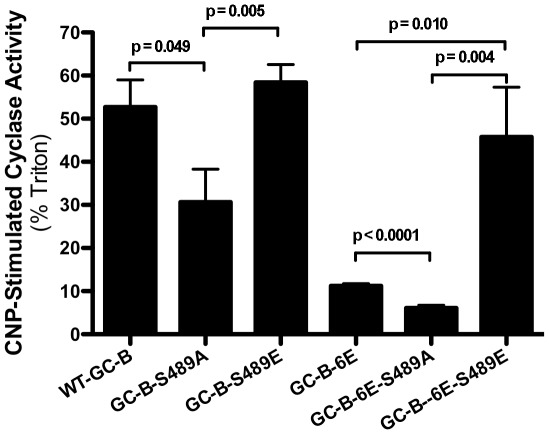
Mutation of Ser-489 in GC-B to alanine but not glutamate decreases CNP-stimulated guanylyl cyclase activity. 293T cells were transiently transfected with WT-GC-B containing alanine or glutamate substitutions for Ser-489 (left side) or with the same substitutions engineered into GC-B-6E (right side). GC assays were conducted for 5 min in the presence of 1 µM CNP, 1 mM ATP and 1 mM magnesium-GTP or 1% Triton X-100 and 5 mM manganese-GTP. GC activity was expressed as CNP-dependent activity/Triton X-100-dependent activity ×100. The results are the mean ± SEM, where n = 8.

### Effect of alanine and glutamate substitutions at Ser-489 in GC-B

Since the mutation of Ser-489 to alanine was the only residue in our initial screen that changed the GC activity ratio without affecting processing, we focused on this residue. First, we characterized the degree of activity loss in fixed substrate concentration GC assays. The CNP-dependent to detergent-dependent GC ratio of S489A-GC-B was reduced to 60% of WT value (Figure 2). In contrast, the activity ratio was insignificantly elevated in membranes from cells transfected with S489E-GC-B where a glutamate was substituted to mimic a receptor constitutively phosphorylated at Ser-489.

Alanine and glutamate substitutions for Ser-489 were also made in a receptor variant containing glutamic acid substitutions at the six previously identified phosphorylation sites (GC-B-6E) [14]. The activity ratio of GC-B-6E was only about 20% of WT GC-B, consistent with the idea that another critical site is not phosphorylated in GC-B-6E. Mutation of Ser-489 to alanine in GC-B-6E decreased the GC activity ratio 45% similarly to how it reduced activity of WT-GC-B-S489A. Surprisingly, mutation of Ser-489 to glutamate in GC-B-6E increased the GC activity ratio 4.1-fold to near WT-GC-B levels.

### Effect of alanine and glutamate substitutions at Ser-473 in GC-A

**Figure 3:**
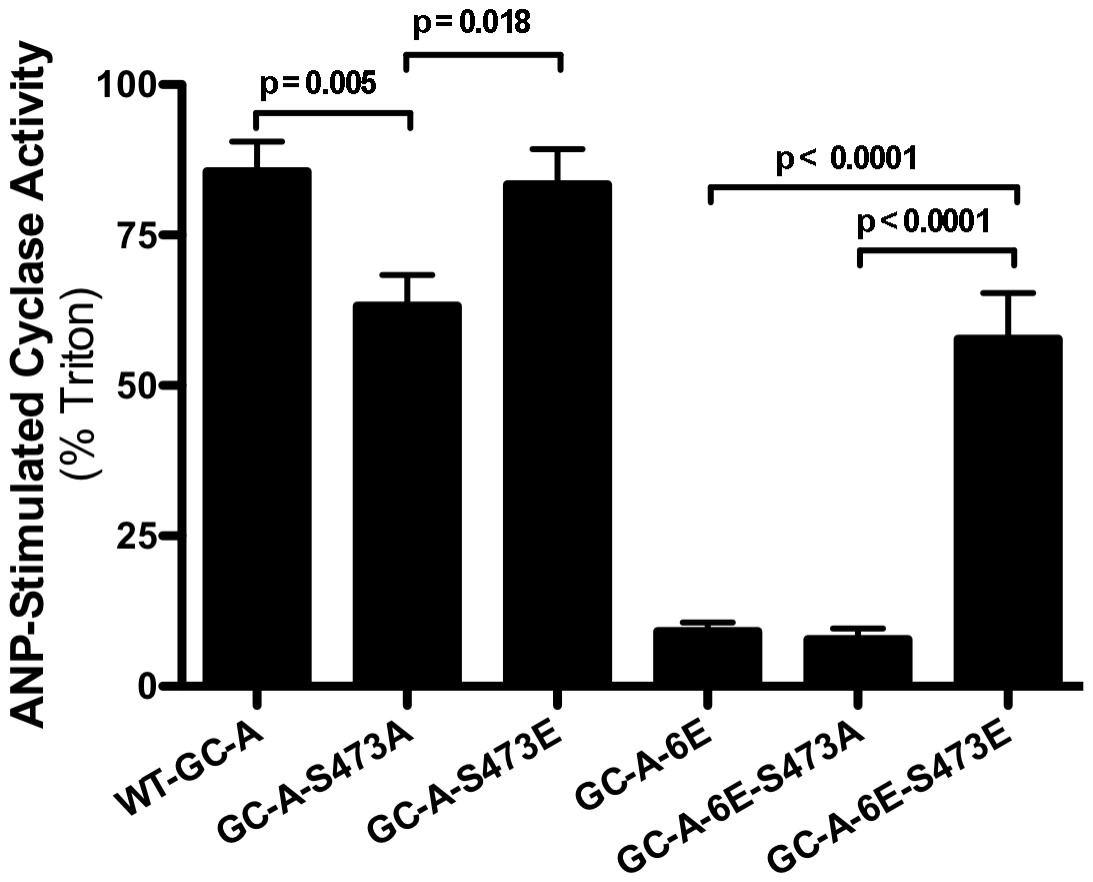
Mutation of Ser-473 in GC-A to alanine but not glutamate decreases ANP-stimulated guanylyl cyclase activity. 293T cells were transiently transfected with WT-GC-A containing alanine or glutamate substitutions for Ser-473 (left side) or with the same substitutions engineered into GC-A-6E (right side). GC assays were conducted in the presence of 1 µM ANP, 1 mM ATP and 5 mM magnesium-GTP or 1% Triton X-100 and 5 mM manganese-GTP. GC activity was expressed as the ANP-dependent activity/Triton X-100-dependent activity ×100. The results are the mean ± SEM, where n = 14.

Since Ser-489 is conserved in all six phosphorylated GCs shown in Figure 1, we investigated the functional consequences of phosphorylation of GC-A at the homologous residue by substituting an alanine or glutamate for Ser-473. As with GC-B, the alanine, but not the glutamate, substitution decreased the natriuretic peptide to detergent GC activity ratio in the WT receptor backbone (Figure 3). Mutations were also made in a receptor containing glutamate substitutions at the original six previously identified phosphorylation sites (GC-A-6E) [15]. The activity ratio of GC-A-6E was low and unlike GC-B-6E, the substitution of alanine for Ser-473 did not result in a statistically significant reduction in activity. However, as with GC-B-6E, substitution of glutamate for Ser-473 in GC-A-6E increased the GC activity ratio (six-fold), restoring activity to near WT levels.

**Figure 4:**
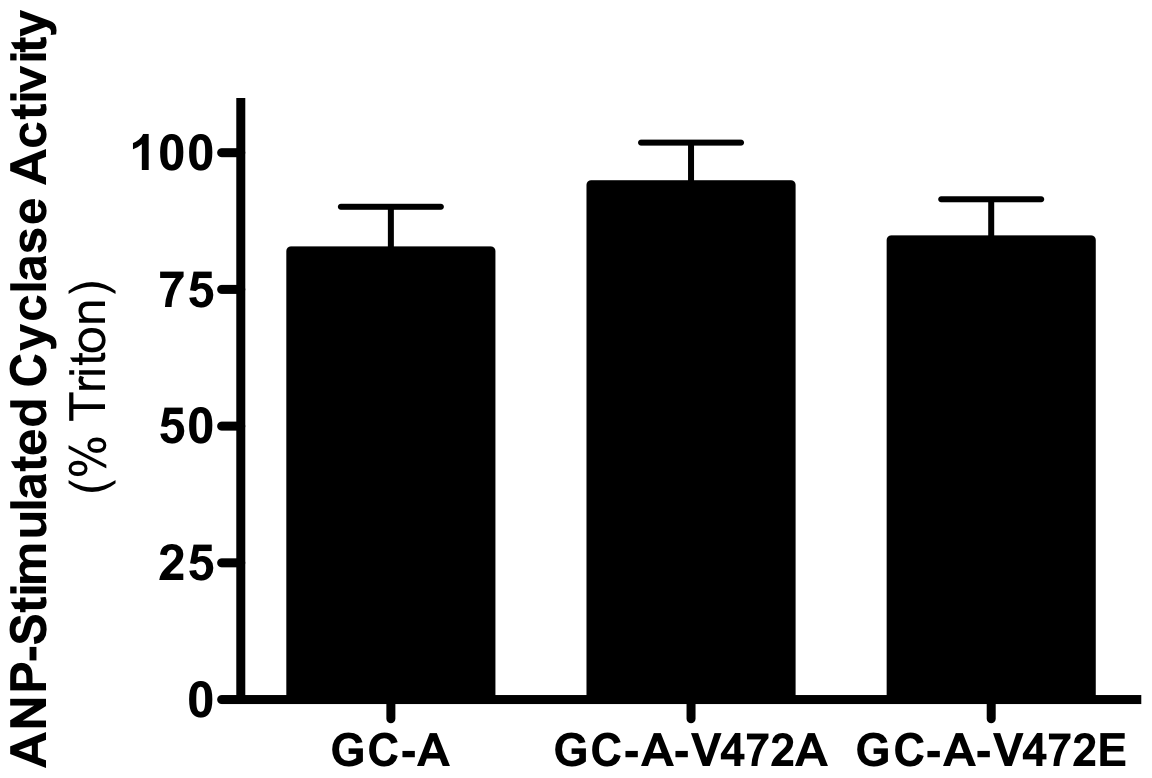
Mutation of Val-472 in GC-A to glutamate or alanine had no effect on ANP-stimulated guanylyl cyclase activity. GC assays were conducted on membrane preparations from 293 cells transiently expressing WT or mutants of rat GC-A as described in figure 2. The results are expressed as the mean ± SEM, where n = 12.

To investigate whether the changes in ligand-stimulated activity associated with Ser-473 in GC-A was specific to that residue or simply a result of increased positive charge in that area of the protein, we mutated the residue N-terminal to Ser-473, Val-472, to alanine or glutamate. Membranes from cells transiently expressing WT or mutant receptors were then assayed for GC activity ratios (Figure 4). Mutation of Val-472 to either amino acid did not alter the activity ratio, which suggest that only specific changes in the charge of the residue at position 473 is sufficient to modulate ANP-dependent activity of GC-A.

### Substitution of GC-B-Ser-489 with alanine but not glutamate reduces the Michaelis constant

**Figure 5:**
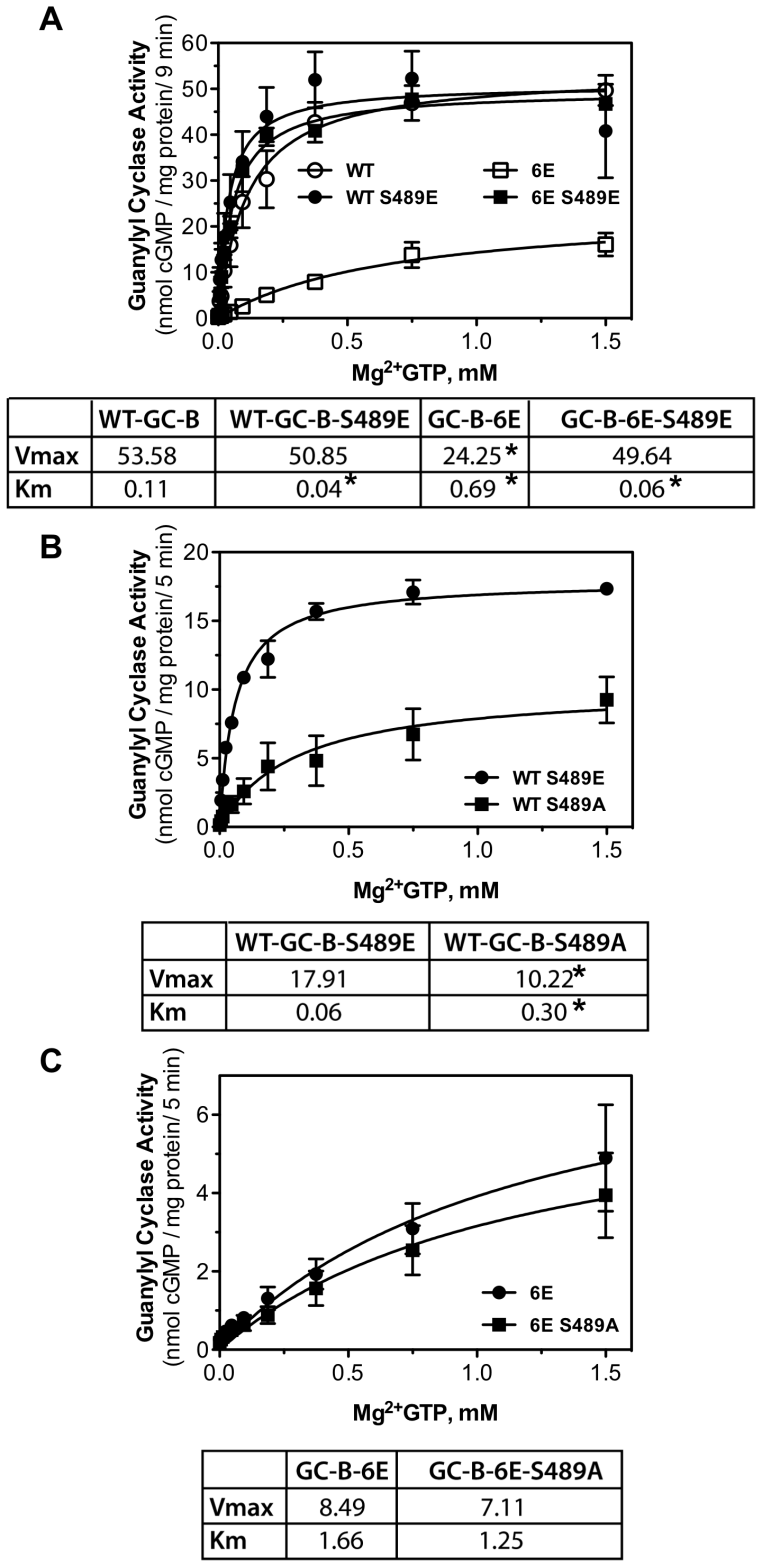
Alanine substitutions at Ser-489 in GC-B increase the Michaelis constant. 293 cells were transiently transfected with the indicated form of GC-B and assayed for GC activity in the presence of 1 µM CNP, 1 mM ATP, and increasing concentrations of GTP. (A) Comparison between WT-GC-B, WT-GC-B-S489E, GC-B-6E and GC-B-6E-S489E. (B) Comparison between WT-GC-B-S489E and WT-GC-B-S489A. (C) Comparison between GC-B-6E and GC-B-6E-S489A. Vmax and Km values were calculated using nonlinear regression. * Indicates significantly different from WT-GC-B (A) or WT-GC-B-S489E (B) at p<0.02.

To determine how the residue at 489 regulates the enzymatic activity of GC-B, we generated substrate-velocity curves for WT-GC-B, WT-GC-B-S489E, GC-B-6E and GC-B-6E-S489E for 9 minutes to determine maximal velocities and Michaelis constants for each enzyme (Figure 5A). We found that maximal velocities for WT-GC-B, WT-GC-B-S489E and GC-B-6E-S489E were unchanged. However, the Vmax for GC-B-6E was two-fold lower and the Km was five-fold higher than the WT enzyme. Unexpectedly, we found that the Michaelis constant for the enzymes containing a glutamate at Ser-489 was lower than that of the WT enzyme. These data suggest that phosphorylation of Ser-489 increases the affinity of the catalytic site for GTP and are consistent with a previous study showing that the Km of GC-A-6E was reduced compared to WT-GC-A [21].

Next, we directly compared substrate-velocity profiles for WT-GC-B-S489E and WT-GC-B-S489A in a shorter five min assay and found that the single substitution of alanine for glutamate increased the Km five-fold and decreased Vmax almost two-fold (Figure 5B). We also compared substrate-velocity profiles for GC-B-6E and GC-B-6E-S489A and found that the Vmax was low and the Km was high for both enzymes and these values were not significantly different, consistent with Ser-489 being dephosphorylated in most molecules of GC-B-6E (Figure 5C).

### Enhanced homologous desensitization of WT-GC-A-S473E and GC-A-6E-S473E

**Figure 6:**
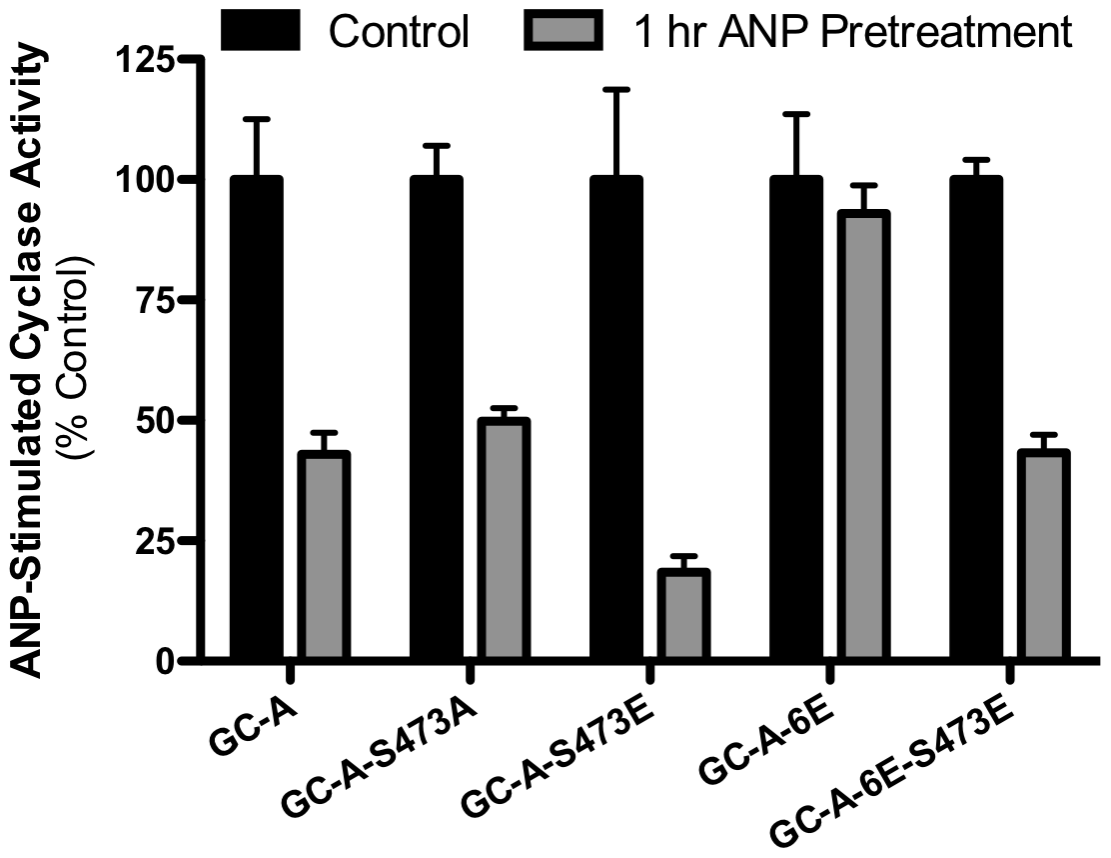
Glutamate substitution for serine 473 enhances homologous desensitization of GC-A. 293 cells transiently expressing WT-GC-A or the indicated mutant forms of GC-A were incubated ±1 µM ANP for 1 hour at 37°C. Membranes were prepared and assayed for GC activity in the presence of ANP, ATP and magnesium-GTP or 1% Triton X-100 and manganese-GTP. The results are expressed as a ratio of hormone-stimulated/detergent-stimulated activity and were normalized to the percent of the activity ratio determined in membranes from cells not exposed to ANP. Data are means determined from multiple experiments ± SEM, where n≥6. * Indicates significantly different from control (GC-A or GC-A-6E) at p<0.01.

Whole 293T cells expressing WT-GC-A, WT-GC-A-S473A, WT-GC-A-S473E, GC-A-6E or GC-A-6E-S473E were incubated in the presence or absence of 1 µM ANP for 1 h at 37°C before membranes were prepared and assayed GC activity in the presence of ANP, ATP and magnesium-GTP or 1% Triton X-100 and manganese-GTP. The data were presented as the ratio of the hormone/detergent activities to control for differences in transfection efficiencies and activity losses resulting from GC-A degradation (Figure 6). Prior ANP exposure decreased ANP-dependent guanylyl cyclase activity of WT-GC-A to 45% of control values, and the desensitization was comparable between the WT-GC-A and GC-A-S473A. However, the activity ratio of the GC-A-S473E mutant receptor was reduced to 20% of initial activity ratio, which is more than double the activity loss observed for WT-GC-A or GC-A-S473A. The effect of the glutamate substitution at serine 473 was also evident when assessed in a receptor backbone containing glutamate substitutions for the six originally identified GC-A phosphorylation sites (GC-A-6E-S473E). Consistent with the desensitization by dephosphorylation hypothesis, homologous desensitization was blunted with GC-A-6E that cannot be dephosphorylated at the six originally identified sites [15]. However, the activity of GC-A-6E-S473E was reduced to 46% or initial values by prior ANP exposure – a much greater desensitization response than observed for GC-A-6E. These data suggest that phosphorylation of Ser-473 may facilitate the homologous desensitization of GC-A.

### GC-B-6E-S489E is resistant to inhibition by calcium and protein kinase C

**Figure 7:**
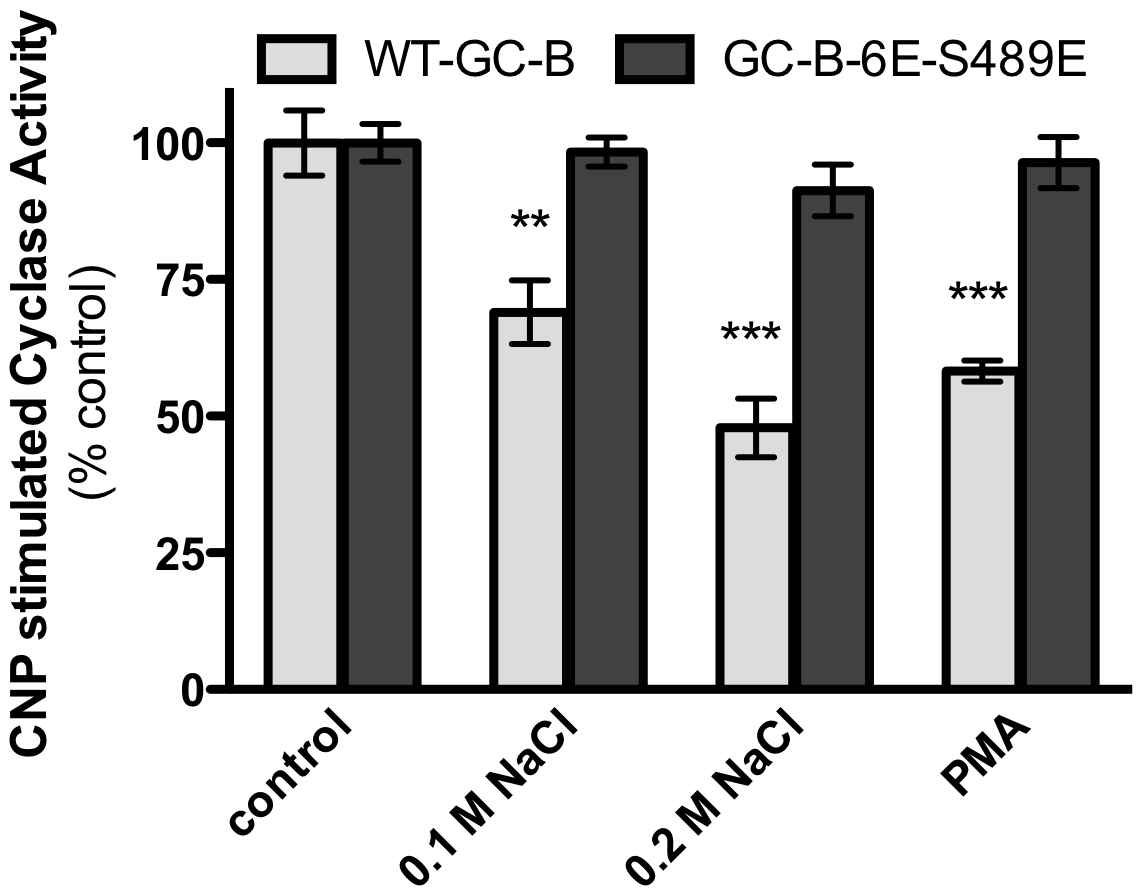
GC-B-6E-489E is resistant to inhibition by hyperosmotic medium and protein kinase C. 293 cells transiently expressing WT-GC-B or GCB-6E-S489E were incubated ±0.1 M NaCl, 0.2 M NaCl or 1 µM PMA for 30 min at 37°C. Membranes were prepared and assayed for GC activity in the presence of CNP, ATP and magnesium-GTP or 1% Triton X-100 and manganese-GTP. The results were expressed as a ratio of hormone-stimulated/detergent-stimulated activity and were normalized to the activity ratio determined in membranes from control cells not exposed to any inhibitory agent. Data are presented as means ± SEM, where n = 4. ** Indicates a p value of <0.01, *** indicates a p value of <0.0001.

A previous study showed that lysophosphatidic acid and hyperosmotic conditions inhibit CNP-dependent activation of GC-B in 3T3 and 293 cells by a process that involves calcium-dependent receptor dephosphorylation [22]. Additional studies demonstrated that phorbol ester activation of protein kinase C inhibits GC-B by a process involving increased phosphorylation of Ser-518 and decreased phosphorylation of Ser-523 in GC-B [23]. Since all known phosphorylation sites of GC-B-6E-S489E are mutated to glutamate, we hypothesized that neither pathway would inhibit this receptor. We tested this hypothesis by incubating 293 cells transfected with WT-GC-B or GC-B-6E-S489E with or without medium containing 0.1 M additional NaCl, 0.2 M additional NaCl or 1 µM phorbol myristic acid (PMA) for 0.5 h before preparing membranes and assaying them guanylyl cyclase activity (Figure 7). Elevated extracellular osmolarity inhibited WT-GC-B in a concentration-dependent manner but failed to reduce the activity ratio of GC-B-6E-S489E. Similarly, PMA exposure inhibited WT-GC-B but did not reduce activity of GC-B-6E-S489E. These data indicate that dephosphorylation is required for calcium- and protein kinase C-dependent inactivation of GC-B.

### Lack of evidence for cellular phosphorylation of Ser-489 and Ser-473

Despite the changes in activity observed when Ser-473 or Ser-489 were mutated, the GC results do not prove that this site is phosphorylated in cells. Thus, we attempted to obtain biochemical evidence of phosphorylation of GC-A at serine 473 and GC-B at serine 489 using mass spectrometric detection. Rat GC-A and GC-B were purified from HEK 293 cells by sequential immunoprecipitation-SDS-PAGE fractionation as previously described [14]. Gel slices of each receptor were digested with trypsin and the resulting peptides were extracted. Samples were enriched for phosphopeptides by immobilized metal affinity chromatography (IMAC). The peptide mixtures were submitted to nLC-MS-MS and the resulting mass/charge data was searched against the IPI rat database. However, these experiments did not yield any matches to phosphopeptides containing Ser-473 or Ser-489 despite numerous attempts allowing for up to two missed cleavage sites or when Arg-C was used as the digesting protease to yield a slightly different peptide containing Ser-489 (data not shown).

Additional experiments were performed where only the parent ion corresponding to the tryptic peptide containing phosphorylated serine 473 was selectively monitored (ELVpSELWR, charge state +2, m/z 556.26). This was done to isolate a low intensity signal that may be “buried” among other peptides that ionize more efficiently. However, this approach also failed to yield a match to a tryptic peptide containing phosphorylated Ser-473 (data not shown).

We also attempted to identify phosphorylated versions of Ser-473 or Ser-489 from GC-A or GC-B, respectively, from receptors isolated from 293 or NIH3T3 cells by mixing synthetic versions of the peptides containing Ser-473 (ELVpSELWR) or Ser-489 (ELApSMLWR) with ^32^PO_4_ labeled peptides isolated from tryptic digests of GC-A and GC-B from metabolically labeled cells as previously described [11]. The ^32^PO_4_–labeled phosphopeptides were identified by autoradiography and the synthetic peptides were visualized by ninhydrin staining. However, no ^32^P-labeled material comigrated with the synthetic peptides (data not shown).

### Poor mass spectrometric detection of the phospho-Ser-473-containing peptide

**Figure 8:**
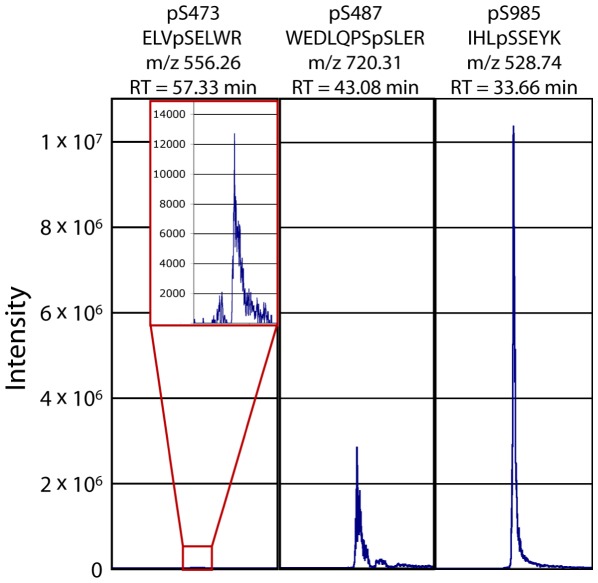
The GC-A tryptic peptide phosphorylated at Ser-473 is poorly detected by mass spectrometry. One pmol of synthetic peptides corresponding to tryptic peptides containing phosphorylated versions of Ser-473, Ser-487, or Ser-985 in GC-A were mixed and submitted to nLC-MS-MS. The resulting ion signal intensities are represented above by their individual m/z ion traces on a standard intensity scale. The inset shown for the phosphor-Ser-473 peptide has an expanded y-axis scale.

To determine if an intrinsic chemical property of this particular phosphopeptide hinders identification by mass spectrometry, we measured detection of synthetic phosphopeptides. Equimolar quantities of three peptides corresponding to tryptic peptides phosphorylated at Ser-473, Ser-487, and Ser-985 in GC-A (ELVpSELWR, WEDLQPSpSLER, and IHLpSSETK, respectively) were mixed and subjected to nLC-MS-MS. The resulting ionized peptide signal intensity for each peptide is shown in Figure 8. The synthetic tryptic peptide corresponding to phosphorylated serine 473 had an intensity 230-fold and 1300-fold less than those corresponding to phosphorylated Ser-487 and Ser-985, respectively. This enormous disparity of signal intensity points to an intrinsic property of the phosphopeptide that inhibits detection of this phosphopeptide by nLC-MS-MS. The reduced sensitivity may result from poor ionization, which is known to be non-uniform for peptides.

## Discussion

By comparing sequences from several phosphorylated GCs, we identified a highly conserved serine nine residues past the membrane spanning regions of GC-A and GC-B. The conserved nature of this serine within a family of proteins that are phosphorylated on multiple serines is consistent with this residue being phosphorylated in both enzymes.

The effect of the amino acid at position 473 in GC-A or position 489 in GC-B on enzymatic activity is striking. When these serines are phosphorylated as expected for most WT enzymes, the Km is around 0.1 mM, which is in the range of cellular GTP concentrations. However, when this single amino acid is mutated to alanine to mimic a constitutively dephosphorylated residue, the Km increases dramatically. Conversely, conversion to glutamate to mimic a constitutively phosphorylated residue “rescues” the enzyme. In fact, the Km for GC-B mutants containing glutamate substitutions for Ser-489 is slightly lower than the Km for the WT enzyme, which suggests that within the WT population most receptors are phosphorylated on Ser-489 but some receptors are dephosphorylated at Ser-489. The fact that the Michaelis constants for GC-B-6E, GC-B-6E-S489A and WT-GC-B-S489A are high but the Michaelis constants for GC-B-6E-S489E and WT-GC-B-S489E are similar to or lower than the WT enzyme strongly suggests that Ser-489 is phosphorylated on most WT-GC-B molecules isolated from 293 cells. Thus, we believe the functional evidence for phosphorylation of Ser-473 in GC-A and Ser-489 in GC-B is compelling. However, we cannot rule out the possibility that the changes in activity are simply coincidental.

Previous studies on enzymatically dephosphorylated preparations of GC-A indicated that dephosphorylation reduced Vmax but not Km (Lincoln Potter, Ph.D. dissertation, Vanderbilt University). Initial studies on GC-A-6E indicated that unlike the mutant containing alanine substitutions for these sites, this receptor was responsive to ANP [15]. Furthermore, GC-A-6E is resistant to ANP-dependent desensitization [15]. Dephosphorylation of GC-B resulting from cellular phorbol ester exposure increased the Km of GC-B about three-fold, whereas dephosphorylation associated with ionomycin-dependent increases in intracellular calcium concentrations primarily reduced Vmax [24]. However, these dephosphorylation events are associated with changes in multiple phosphorylation sites, not a single site as investigated in this study [23], [24]. The large increase in the Km resulting from a single alanine substitution reported here was unexpected and provides impetus for systematic experiments examining the effect of alanine and glutamate substitutions for all known phosphorylation sites on the Vmax and Km of GC-A and GC-B.

We were unable to obtain biochemical proof of phospho-Ser-473 or phospho-Ser-489 from cells using mass spectrometric or ^32^PO_4_-tryptic phosphopeptide mapping approaches. However, this does not prove that these sites are not phosphorylated in physiologic systems since no current method of phosphorylation site identification is infallible. This is illustrated by the progression of phosphorylation site discovery in GC-A and GC-B. Metabolic labeling studies using ^32^P-orthophosphate in conjunction with phosphopeptide mapping techniques initially revealed six sites in GC-A and five sites in GC-B [11], [12]. These studies noted that ∼90% of the total counts initially incorporated into the receptor were lost during sample preparation prior to visualization and suggested the distinct possibility that endogenous phosphorylation sites were missed by this technique. Recent mass spectrometry studies, which identified novel phosphorylation sites at Ser-487 in GC-A and Thr-529 in GC-B, proved that this was the case [13], [14].

Similarly, mass spectrometry is not absolute in its detection of all endogenous phosphorylation sites. For example, we were unable to detect endogenous phosphorylation at serine 510 and threonine 513 in the human GC-A receptor using a mass spectrometric approach. There are several explanations for why an endogenous phosphorylation site may not be detected by a mass spectrometry approach. First, the chemistry of the tryptic phosphopeptide containing the site of interest may be such that it is lost during sample preparation prior to injection on the instrumentation, *e.g.* it preferentially adheres to the sample tube. Once injected into the instrument, the chemical properties may be such that it does not ionized well, and as a result, very little enters the detector. Alternatively, a poorly ionized peptide can easily be “buried” if several other peptides with higher intensities have overlapping retention times because the instrument preferentially picks the ions with the highest intensities to submit for tandem mass spectrometry. Unfortunately, the tryptic phosphopeptide corresponding to a phosphorylated Ser-473 suffers from poor detection issues. Thus, we were unable to validate that Ser-473 or Ser-489 are phosphorylated in GC-A or GC-B, respectively, possibly due to poor detection of the peptide by the current techniques.

In conclusion, we identified a highly conserved serine in GC-A and GC-B that decrease natriuretic peptide-dependent GC activity when mutated to an alanine but not when mutated to glutamate as predicted for a phosphorylation site. We also found that the conversion of this highly conserved residue to a glutamate increases the activity of GC-A-6E and GC-B-6E to near WT levels. Importantly, we determined that that the phosphorylation of this single site dramatically reduced the Michaelis constant of GC-B and increased the magnitude of the homologous desensitization response for GC-A. Whether these residues are *bona fide* phosphorylation sites awaits chemical identification of peptides containing these sites from biological samples. However, the marked effect of the phosphomimetic mutations on the kinetic and desensitization properties of the enzymes provide compelling support for the hypothesis that Ser-489 in GC-B and Ser473 in GC-A are phosphorylated in physiologic systems.

Finally, these studies are important in terms of designing receptor constructs for genetic “knock in” or “transgenic” experiments used to investigate the role of phosphorylation and dephosphorylation in the regulation of these receptors *in vivo*. The similarity of the kinetic parameters of GC-B-7E to WT-GC-B make the receptor containing the glutamate substitution for Ser-489 an excellent choice for future genetic replacement studies because this receptor is resistant to inhibition and has a Km that is similar to the WT receptor [25].

## Materials and Methods

### Reagents

[^32^P]-α-GTP and ^125^I-cGMP radioimmunoassay kits were purchased from Perkin Elmer Life Sciences (Waltham, MA). PhosSelect affinity gel for immobilized metal affinity chromatography (IMAC), trypsin, creatine kinase, phorbol myristic acid and rat ANP and CNP were from Sigma-Aldrich (St. Louis, MO). Protease inhibitor cocktail was from Roche Applied Bioscience (Indianapolis, IN).

### Cells and transfections

293 neo, 293T- rat GC-A and 293T- rat GC-B cells were maintained as previously described [26]. The 3T3 cells stably expressing GC-A and GC-B were maintained as previously described [8], [19]. Approximately 60% confluent 293neo cells grown on polylysine coated 10 cm plates were transfected with 5 µg of plasmid DNA using the HEPES-calcium-phosphate precipitation method. Transfection efficiency was ∼80% based on GFP expression. The medium was replaced after 3 h and membranes were harvested 48 h after transfection.

### Mutagenesis

Site-directed mutagenesis of rat GC-A and GC-B was performed using the QuikChange II system (Stratagene, Cedar Creek, TX) as previously described [27]. All mutations were confirmed by DNA sequencing.

### Membrane preparation

Crude membranes were prepared at 4°C in phosphatase inhibitor buffer which consisted of 50 mM 4-(2-hydroxyethyl)-1-piperazineethanesulfonic acid – pH 7.4, 50 mM NaCl, 20% glycerol, 50 mM NaF, 1 mM EDTA, 0.5 µM microcystin and 1X Roche protease inhibitor cocktail.

### Guanylyl cyclase assays

Single substrate concentration GC assays were performed at 37°C for 5 min in a buffer containing 25 mM 4-(2-hydroxyethyl)-1-piperazineethanesulfonic acid – pH 7.4, 50 mM NaCl, 0.1% BSA, 0.5 mM 1-methyl-3-isobutylxanthine, 1 mM GTP, 0.5 µM microcystin, 1 mM EDTA, and 1–2 µCi of [α-^32^P] GTP, with either 5 mM MgCl_2_, 1 mM ATP, and 1 µM natriuretic peptide or 1% Triton X-100 with 5 mM MnCl_2_ being substituted for the MgCl_2_. Reactions were started by the addition of 0.08 ml of the above reagents to 50–200 µg of crude membrane protein suspended in 0.02 ml of phosphatase inhibitor buffer. Reactions were stopped by the addition of 0.5 ml 110 mM ZnOAc and 0.5 ml 110 mM NaCO_3_ on ice. ^32^P-cGMP was purified by alumina column chromatography and detected by the Cerenkov method in a gamma counter.

Substrate-velocity experiments were conducted for 5 or 9 min as previously described [28]. Reactions were stopped with 0.4 ml of ice-cold 50 mM sodium acetate buffer containing 5 mM EDTA. Cyclic GMP concentrations were determined by radioimmunoassay. Because enzymatic activity was not completely linear with time, we qualify the kinetic parameters obtained under these conditions as “apparent.”

GC activities in membranes from transfected 293 cells were at least 50-fold higher than activities in membranes from untransfected cells, which indicated that the GC activity was due to the transfected enzyme. We previously demonstrated that GC-A and GC-B are regulated similarly in 293 cells as they are in endogenously expressing cells [21], [29].

### Mass spectrometric detection

Receptor purification by immunoprecipitation, in-gel digestion, phosphopeptide separation and mass spectrometric detection were performed as we previously described [14].

### ^32^PO_4_-phosphopeptide mapping

Tryptic phosphopeptide mapping comigration studies were conducted as we previously described for GC-B [11].

### Statistical analysis

All single substrate GC assays were evaluated using an unpaired t-test where p≤0.05 was considered significant. Values were presented as mean ± SEM. Vertical bars within the columns represent SEM. Brackets indicate t-test between the indicated values. Substrate-velocity curves were analyzed by non-linear regression using an a Michaelis-Menten model to determine Vmax and Km. Significant differences between nonlinear regression curves used to determine Km and Vmax values were determined using the extra sum of squares F test to generate p-values.

## Supporting Information

Figure S1 Effect of serine or threonine to alanine substitutions on the processing and expression of wild type and mutant forms of GC-B. 293 cells were transiently transfected with the indicated GC-B constructs and Western blotting of immunoprecipitated receptors assessed the expression and processing of the receptor. Immunocomplexes were fractionated on an 8% SDS reducing gel and blotted to an Immobilon membrane, which was probed with rabbit polyclonal antiserum 6328 against the C-terminus of GC-B. Detection was by horseradish peroxidase conjugated secondary antibody ECL. WT-GC-B consists of a thin, deglycosylated fastest migrating species, a partially glycosylated slower migrating species, and fully glycosylated and phosphorylated slowest migrating species (arrow) that is hormonally responsive. Mutation of Ser-647 caused the loss of the upper fully processed form of GC-B.
(TIF)
