# Supplementary figures and images for: A Functional Screen Provides Evidence for a Conserved, Regulatory, Juxtamembrane Phosphorylation Site in Guanylyl Cyclase A and B
Source: PLoS One. 2012 May 9;7(5):e36747. doi: 10.1371/journal.pone.0036747 (PMC3348905; doi:10.1371/journal.pone.0036747)

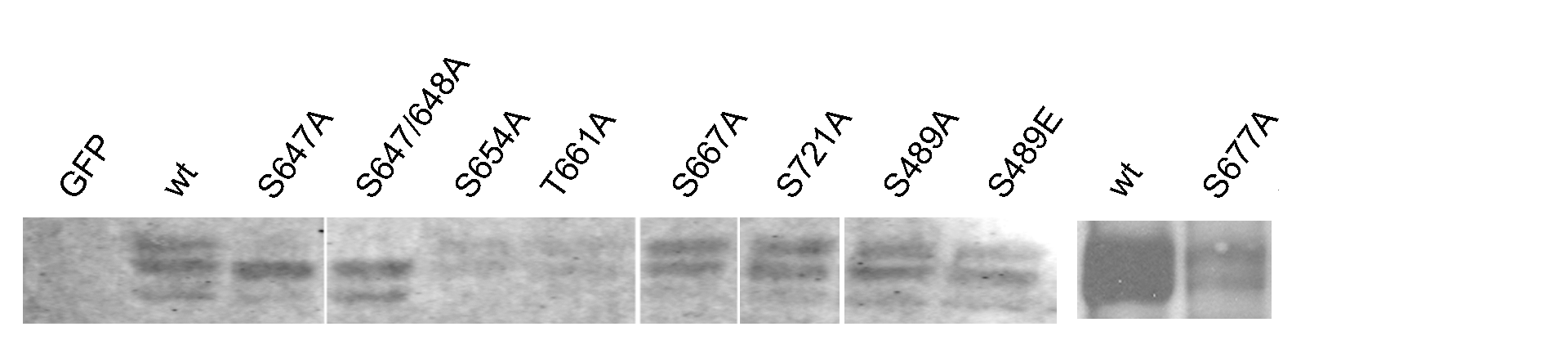

Supplement: Figure S1 — Effect of serine or threonine to alanine substitutions on the processing and expression of wild type and mutant forms of GC-B. 293 cells were transiently transfected with the indicated GC-B constructs and Western blotting of immunoprecipitated receptors assessed the expression and processing of the receptor. Immunocomplexes were fractionated on an 8% SDS reducing gel and blotted to an Immobilon membrane, which was probed with rabbit polyclonal antiserum 6328 against the C-terminus of GC-B. Detection was by horseradish peroxidase conjugated secondary antibody ECL. WT-GC-B consists of a thin, deglycosylated fastest migrating species, a partially glycosylated slower migrating species, and fully glycosylated and phosphorylated slowest migrating species (arrow) that is hormonally responsive. Mutation of Ser-647 caused the loss of the upper fully processed form of GC-B. (TIF) [file pone.0036747.s001.tif]
